# Small and long RNA transcriptome of whole human cerebrospinal fluid and serum as compared to their extracellular vesicle fractions reveal profound differences in expression patterns and impacts on biological processes

**DOI:** 10.1186/s12967-022-03612-3

**Published:** 2022-09-08

**Authors:** Uwe Michel, Orr Shomroni, Barbara Müller, Peter Lange, Gabriela Salinas, Mathias Bähr, Jan Christoph Koch

**Affiliations:** 1 Department of Neurology, University Medical Center Göttingen, Robert-Koch-Straße 40, 37075 Göttingen, Germany; 2 Evotec International GmbH, Marie-Curie-Str. 7, 37079 Göttingen, Germany; 3 Institut Für Humangenetik, NGS-Integrative Genomics (NIG), University Medical Center Göttingen (UMG), Justus-von-Liebig Weg 11, 37077 Göttingen, Germany

**Keywords:** Human cerebrospinal fluid, Human serum, Extracellular vesicles, Small and long RNA transcriptome, RNA expression patterns

## Abstract

**Background:**

Next generation sequencing (NGS) of human specimen is expected to improve prognosis and diagnosis of human diseases, but its sensitivity urges for well-defined sampling and standardized protocols in order to avoid error-prone conclusions.

**Methods:**

In this study, large volumes of pooled human cerebrospinal fluid (CSF) were used to prepare RNA from human CSF-derived extracellular vesicles (EV) and from whole CSF, as well as from whole human serum and serum-derived EV. In all four fractions small and long coding and non-coding RNA expression was analyzed with NGS and transcriptome analyses.

**Results:**

We show, that the source of sampling has a large impact on the acquired NGS pattern, and differences between small RNA fractions are more distinct than differences between long RNA fractions. The highest percentual discrepancy between small RNA fractions and the second highest difference between long RNA fractions is seen in the comparison of CSF-derived EV and whole CSF. Differences between miR (microRNA) and mRNA fractions of EV and the respective whole body fluid have the potential to affect different cellular and biological processes. I.e. a comparison of miR in both CSF fractions reveals that miR from EV target four transcripts sets involved in neurobiological processes, whereas eight others, also involved in neurobiological processes are targeted by miR found in whole CSF only. Likewise, three mRNAs sets derived from CSF-derived EV are associated with neurobiological and six sets with mitochondrial metabolism, whereas no such mRNA transcript sets are found in the whole CSF fraction. We show that trace amounts of blood-derived contaminations of CSF can bias RNA-based CSF diagnostics.

**Conclusions:**

This study shows that the composition of small and long RNA differ significantly between whole body fluid and its respective EV fraction and thus can affect different cellular and molecular functions. Trace amounts of blood-derived contaminations of CSF can bias CSF analysis. This has to be considered for a meaningful RNA-based diagnostics. Our data imply a transport of EV from serum to CSF across the blood–brain barrier.

**Supplementary Information:**

The online version contains supplementary material available at 10.1186/s12967-022-03612-3.

## Background

In the coming decades aging populations will cause an increased number of people spending more lifetime with disabling neurodegenerative diseases like dementia and Parkinson’s disease [1]. Therefore, medical treatments and appropriate diagnostic tools are urgently needed to maintain health-related quality of life of elderly people and also to minimize the ethical and financial burden for societies. Therefore, in recent years many efforts were taken to improve early diagnosis of initial pathological changes in neurodegenerative diseases, as this is a precondition to interfere with disease progression before obvious and often irreversible clinical symptoms appear.

Cerebrospinal fluid (CSF) from lumbar punctures is frequently used for biomolecule-based diagnostics of neurological diseases, and combinations of marker molecules were proven useful for the diagnosis of neurodegenerative, inflammatory and infectious diseases of the central nervous system (CNS). Nevertheless, none of the biomarkers currently in use is exclusively specific for only one disease condition, and the diagnostic value of proteins, DNA and other marker molecules still depend on additional diagnostic findings and the knowledge of the clinical context [2–6]. This deficiency, and the above mentioned pressing need to face the expected increase in neurodegenerative diseases, urge for the discovery and validation of reliable, specific, and prognostic marker molecules.

In the last decade, the detection and characterization of RNA species in different body fluids reflecting the transcriptome of their sources of origin, has fuelled hope for the development of new, specific prognostic and diagnostic RNA-markers [7, 8]. But very recent work summarizing the state of the art of RNA-based diagnostics clearly points out that current achievements in this field cannot yet live up to the initial expectations [9–13]. A lack of standardized workflows from sample generation to RNA-extraction and finally to RNA-measurement is the main reason for this deficiency. Comparisons between studies are still hampered by differences in sample collection [14], sample processing [7, 9, 14–17], technical variability in RNA-profiling platforms [9, 18–22] and RNA analysing algorithms [23], variability between technical replicates [24], studies with small sample sizes that disregard rare RNAs with low detection limits [7, 16, 18, 25, 26], biased results through sample contamination with blood-derived cells [14, 20, 27, 28], and studies with small numbers of cases and thus low statistical power [18, 26].

In addition to the described hindrances, more general challenges connected to human samples like CSF need to be considered, i.e., the total volume of CSF in adults is approximately 150 ml, and has an average, age-dependent turnover rate of approximately four times a day that depends on the physical activity [29]. This, and gender- and age-dependent differences, seen in RNA-analysis of CSF [14, 30] partially explain the observed donor-to-donor variations, and similar considerations come true for blood samples as well [24, 31, 32]. Further confounding factors are the genetic heterogeneity, medication, a high variability in RNA-turnover [27], and RNA-concentrations of CSF which usually are below the limit of detection of most methods [19, 22, 26, 27]. Furthermore, certain RNA species can selectively be packed in extracellular vesicles (EV) [25, 30, 33–35], whereas others seem mainly bound to extravesicular proteins [24, 25, 32, 36–39]. The latter raises the question whether RNA extracted from CSF-fractions, or total RNA from whole CSF is best suited for disease prognosis and diagnosis, or whether profiles of both fractions are necessary to comprehend all RNA-associated characteristics of a disease. In order to address this crucial question, we prepared and analyzed RNA from EV and total RNA from large volumes of pooled human CSF samples and analyzed both with next-generation-sequencing (NGS).

Here we show that body fluids and their respective EV have significantly different compositions of long and small RNA, and that miR derived from whole body fluid and respective EV have the potential to affect different cellular and biological processes.

## Materials and methods

### Collection of human CSF

CSF samples were collected according to clinical necessities for routine diagnostics in the Department of Neurology, University Medical Center of Göttingen. Only samples from patients who presented in the clinic with a variety of symptoms but finally had no obvious signs of a known disease were included in this study. Before onsets of clinical routine diagnostics, the number of erythrocytes and leucocytes were counted manually in each sample, and within one hour after aspiration the samples were centrifuged for ten minutes at 105 × g. Cell-free supernatants were carefully aspirated for further clinical analysis. Samples with signs of haemolysis before the centrifugation step or samples that had counts above 275 erythrocytes/µl before and/or signs of haemolysis after the centrifugation step were excluded from this part of the study. Furthermore, only samples with leucocyte counts below 8 cells/µl were included in this part of the study. Five samples with leucocyte counts from five to eight leucocytes/µl before centrifugation were also made cell-free by centrifugation and were also included in this part of the study, as their donors had no obvious signs of a known disease. After completion of clinical analysis, the remnants of the CSF samples were stored frozen at − 80 °C until further processing for this study. No CSF samples were specifically collected and no extra CSF samples were drawn from any of the patients for the purpose of our research. No identifying information was acquired for this study, and patients gave prior written consent to the scientific use of their samples. For this part of the study 324 CSF samples were collected, 161 samples (49,69%) from male and 163 (50,31%) samples from female patients. The average age of the patients was 55,5 ± 21,1 years; for more detailed information about the samples, please refer to Additional file 1: Fig. S1A–C.

For the purpose of comparison, samples were also collected from CSF with high erythrocyte counts before the 105 × g centrifugation step, or from CSF with obvious signs of haemolysis after the lumbar puncture; in none of samples the number of leucocytes was above 23 before the centrifugation step. These samples were processed completely separate from normal CSF and will be referred to further on as blood-contaminated CSF samples. 36 blood-contaminated CSF samples were collected, 21 samples (58,33%) from male and 15 (41,67%) samples from female patients. The average age of the patients was 61,45 ± 23,3 years; for more detailed information about the blood-contaminated samples, please refer to Additional file 1: Fig. S1D–F.

From all CSF samples a 100 µl aliquot was tested for bacterial or fungal contamination for five days at 37 °C in cell culture medium; none of the samples included in the study showed any signs of contamination.

### Processing of human CSF samples

CSF samples necessary to make up a total volume of 74 ml were thawed on ice and pooled. The pooled samples were mixed and briefly centrifuged in order to divide the pool in two equal aliquots of 37 ml. One aliquot was used for column centrifugation and the other one for extracellular vesicle (EV) preparation; 20 aliquots for each kind were prepared.

#### Ultrafiltration of 36 ml aliquots of CSF with spin columns

Ultraspin columns are molecular size-based filters that amongst others can be used to isolate protein-bound RNA and RNA included in extracellular vesicles [38, 40]. Tuchinovic’s work with human plasma and cell culture medium and own work with serum and blood-contaminated CSF samples showed that concentrates from appropriate-sized filters of ultraspin columns retained all of the measurable RNA content, whereas the corresponding filtrates were depleted of measurable amounts of RNA (see Additional file 1: Fig. S2). This work was done with 100 KDa ultraspin columns, but as in our hands 50 KDa columns seemed to be more consistent in respect to the processing time of CSF samples than 100 KDa columns, we accomplished all RNA-preparations of whole CSF for next-generation sequencing (NGS) with 50 KDa ultraspin columns.

Three ml CSF were pipetted into each of four ultrafiltration spin columns with a molecular cut-off of 50 KDa (Vivaspin Turbo4; Satorius, Germany) and centrifuged at 1860 × g at 4 °C until the volume was concentrated to approximately 250 µl. Then to each of the four spin columns another 3 ml of CSF was added, and the columns were again spun until the volumes were concentrated to approximately 250 µl. This step was repeated one more time, but at the last centrifugation the volume was concentrated to approximately 200 µl. The first centrifugation step takes around 10 min, the second about 18 min and the last step approximately 25 min. After centrifugation the four resulting concentrates of the 36 ml CSF were transferred to DNA-low-binding tubes and each emptied concentration chamber of the spin columns was rinsed once with 50 µl ice-cold 10 mM TRIS pH 7, 4. The rinses were then added to the respective concentrates to make up a total volume of 250 µl in each of the four DNA-low-binding tubes; these four CSF-concentrates were finally used for preparation of one RNA sample.

### Extracellular vesicle preparation of 36 ml aliquots of human CSF with ultracentrifugation

11, 2 ml ice-cold PBS was added to 36 ml of pooled CSF samples; the combined volumes were carefully mixed, briefly centrifuged, and divided into four times 11,8 ml, which were distributed to four ultracentrifugation tubes (Beckman coulter). The tubes were balanced with ice-cold PBS and then centrifuged at 4 °C at 180,000 × g for 4 h. The resulting supernatants were aspirated by pipetting and to each pellet 1 ml of Tri-Reagent (Sigma T9424) was added. The tubes were vortexed for 30–60 s, briefly centrifuged and the suspensions were then transferred to 1,5 ml DNA-low-binding tubes. The suspensions were left standing for 5 min at room temperature and then further used for RNA-preparation.

### RNA-preparation of ultrafiltrated CSF

To each of the four 250 µl CSF-ultrafiltrates, 0,75 ml Tri Reagent (Sigma T3934) was added; the mixtures were vortexed for 60 s and then left standing for five minutes at room temperature. Then 100 µl of 1-bromo-3-chloropropane was added to each tube, samples were vortexed for 30 s and left standing at room temperature for five more minutes. Samples were then spun at 12,000 × g at 4 °C for 10 to 15 min to separate the watery from the organic phases. 350 µl from each upper watery phase were transferred to a 2 ml DNA-low-binding tube. The remaining watery phases of the first extractions, were reextracted with 400 µl of 10 mM TRIS pH 7,4 (vortexed for one minute, left standing for five minutes and centrifuged for 10 to 15 min), and 450 µl of the reextracted watery phases were then combined with the 350 µl volumes of the first extraction step. To each sample 4 µl of glycoblue (15 mg/ml) and 27,5 µl 3 M sodium acetate pH 5,2 were added. Samples were mixed carefully and then 800 µl of -20 °C cold isopropanol (equivalent to the volumes of the combined watery phases) were added to each sample; samples were again vortexed and then stored for RNA-precipitation overnight at − 20 °C. The next day one of the four samples was centrifuged for 45 min at 4 °C at 13,000 × g, the supernatant was decanted and the content of another tube from the precipitation step was pipetted onto the pellet of the first tube. The tube was again centrifuged for 45 min at 4 °C at 13,000 × g and the supernatant was decanted; this was repeated until the content of all tubes from the precipitation step were concentrated in one tube, resulting in a pellet that combines the RNA of 36 ml whole CSF. After washing the pellet once with 1000 µl 75% ethanol, it was resuspended in 75% ethanol and kept at -80 °C until to the last precipitation step. In the last step the RNA was pelleted by centrifugation for 45 min at 4 °C at 13,000 × g and each pellet was dissolved in 8 µl 10 mM TRIS pH 7,4 for NGS analysis.

### RNA-preparation of extracellular vesicles from CSF

The four tubes containing the 1 ml Tri-Reagent and the extracellular vesicle RNA were then treated as described in *Extracellular vesicle preparation of 36 ml aliquots of human CSF with ultracentrifugation.* Then 100 µl 1-Bromo-3-Chlor-Propane were added to each tube and the suspensions were vortex for 15 s. Tubes were again left standing for five minutes before they were then treated as described for RNA-preparation from ultrafiltrated CSF (see above).

### Collection and processing of human serum samples

Sixteen samples of 15 ml of blood were collected from healthy volunteers of our research group and volunteers who donated blood to the blood bank of the University Medical Center of Göttingen. Serum was separated with serum separator tubes at 2000 × g for ten minutes at 4 °C. After centrifugation the serum was aliquoted and stored frozen at -80 °C. A 100 µl aliquot of each serum sample was used to determine the hemoglobin content, and only samples with hemoglobin concentrations below the limit of detection of the routine analysis (< 5 mg/dl) were used for further processing. For more detailed information about the age and sex distribution of the sample donors, please refer to Additional file 1: Fig. S3.

### RNA-preparation of whole serum

The preparation of RNA from serum concentrates of ultraspin columns is hampered by an approximately 200fold higher protein concentration in serum as compared to CSF; this results in long centrifugation times and extremely viscous concentrates that are difficult to pipette. Therefore, total RNA of serum was only prepared by ultraspin columns to proof the principle of the method, whereas total RNA of serum for NGS was exclusively extracted with Tri-Reagent (Tri-Reagent BD (T3809) for blood). For this purpose, 1 ml human serum samples from six donors were thawed on ice-water. From each single 1 ml sample four aliquots of 250 µl were added to four 1,5 ml DNA-low-binging tubes containing 750 µl Tri-Reagent. The tubes were vortex for 30 to 60 s and left at room temperature for five minutes, then 100 µl 1-bromo-3-chloropropan were added to each tube and the mixtures were again briefly vortexed and incubated for five minutes at room temperature. Then the RNA was prepared from each sample exactly as described in *RNA-preparation of ultrafiltrated CSF,* resulting in four independent RNA preparations that finally were pooled to one RNA sample.

### RNA-preparation of extracellular vesicles from serum

One millilitre of serum was added to 9 ml of ice-cold PBS in ultracentrifugation tubes, the tubes were carefully mixed and briefly centrifuged to collect all liquid; tubes were then balanced with ice-cold PBS and centrifuged for 4 h at 180,000 × g at 4 °C. After centrifugation the supernatants were carefully pipetted from the pellets and 1 ml Tri-Reagent was added to each tube. In contrast to the extracellular vesicle pellets of CSF, the extracellular vesicles of serum formed visible pellets, and the resuspension of these pellets was achieved by vortexing and holding the tubes briefly in an ice-cold ultrasonic bath. After resuspension and five minutes incubation at room temperature, 100 µl 1-bromo-3-chloropropan were added to each tube, and then samples were exactly treated as described for RNA-preparation from ultrafiltrated CSF.

### MiR- and mRNA-sequencing and transcriptome analysis

The non-coding RNA sequencing (ncRNA-seq) and its primary analysis were performed by the NGS Integrative Genomics Core Unit (NIG, Göttingen, Germany). For RNA-sequencing RNA samples were subjected to non-stranded mRNA library preparation using the TruSeq RNA Sample Prep Kit v2 with minor modifications (ligation and PCR amplification cycles). Fragment sizing of final libraries were analyzed via Fragment Analyzer (average of 300 bp). Libraries were sequenced (SE, 30 Mio reads/sample)) on the HiSeq 4000 platform. For miR library preparation we used the QIAseq miR Library Kit, a gel-free miR sample according to manufacture recommendations. Fragment sizing of final libraries were analyzed via Fragment Analyzer (average of 70 bp). Libraries were sequenced (SE,10 Mio reads/sample)) on the HiSeq 4000 platform.

The whole RNA from each sample was used for both, the small and long RNA NGS approach. Sequenced reads were initially trimmed for Qiagen Small RNA 3’ Adapter using cutadapt version 2.10 [41]. The trimmed reads were aligned to the Homo sapiens non-coding regions in hg38 from ENSEMBL (https://www.ensembl.org/Homo_sapiens/Info/Index) using bowtie2 version 2.3.4 with default parameters [42]. High-quality mapped reads (MAPQ = 1 or MAPQ > 4) were selected from the resulting alignment files and quantified for the non-coding regions in the Homo sapiens sapiens genome assembly hg38 using Salmon version 1.2.1 [43] using traditional expectation maximization (EM) algorithm. Finally, deregulated non-coding RNAs were derived by comparing samples from various conditions (e.g. whole CSF vs. CSF EV and whole serum vs. serum EV) using the R package DESeq2 version 1.31.5 [44], where the initial filtering condition involved RNAs with ≥ 10 counts per RNA species in at least one sample of each group was kept.

Sequenced reads of long RNA were aligned to the Homo sapiens sapiens genome assembly hg38 from ENSEMBL (https://www.ensembl.org/Homo_sapiens/Info/Index) using STAR version 2.5.2 with default parameters [45]. The resulting alignment files were used to quantify the number of reads per gene in human gene assembly version 97 using featureCounts version 1.5.0 [46]. Similarly to the non-coding RNAs, transcripts were analysed for their deregulation between various conditions using the R package DESeq2 and relying on the same filtering (RNA must have ≥ 10 counts in at least one sample of each group).

While miR could be tested for deregulation between particular conditions, determining their biological context was more challenging, since direct association of miR and functional terms (gene ontology categories or pathways) were not available, thus making a direct enrichment analysis of biological terms impossible. Therefore, the analysis involved initially annotating the miR to their target coding genes, and then using those target genes for the enrichment analysis. In brief, all transcripts tested for a particular comparison (e.g. whole CSF vs. CSF-derived EV) were overlayed with their ENSEMBL gene IDs from the human gene set version 97 (http://ftp.ensembl.org/pub/release-97/gtf/homo_sapiens/Homo_sapiens.GRCh38.97.gtf.gz). The ENSEMBL gene IDs were mapped to their comparable miRBase IDs using the R package biomaRt. Utilizing the miRBase IDs of miR of interest as input, the R package multiMiR was used to extract target genes of those miR, where validated targets relied on the databases miRecords [47], miRTarBase [48]and TarBase [49]. Finally, an over-representation-analysis (ORA) was performed using WebGestalt [50], where the target genes of particular sets of deregulated miR were used as input, and the target genes of all miR tested in a particular differential expression analysis were used as the reference.

Samples derived from low quality libraries in which the number of detected RNA-species was more than 4.9 fold standard deviations below the mean of all RNA-species found in quality libraries, were excluded from further evaluation. From long RNA-sequencing four libraries had to be excluded (two libraries from the CSF-EV-, one from the whole CSF- and one from the serum EV-fraction); from small RNA-sequencing all libraries were included in the analysis. Statistical differences between groups were analyzed with the Mann Whitney test with Prism 7 for Mac.

## Results

### General results

The time necessary for routine analysis of CSF depends on clinical requirements; i.e., some CSF samples can be analysed within hours, whereas several days might be necessary for more detailed analyses. Therefore, CSF samples have to be kept at 4 °C for varying periods until the remnants can be stored at − 80 °C. We thus analyzed the effect of long-time storage of CSF at 4 °C on the RNA content. CSF samples were divided in 2 equal aliquots, one was directly stored frozen at − 80 °C after clinical analysis and the second was left for 14 days at 4 °C; RNA from these parallel aliquots was then isolated with spin columns as described. Additional file 1: Fig. S4A shows that the RNA-content of both groups did not differ significantly from each other. In addition, treatment of CSF concentrates from 100 KDa columns with RNAse also had no obvious effect on the recovery of RNA from human CSF (Additional file 1: Fig. S4B), supporting observations, which suggest that RNAs in body fluids are largely protected by EV and RNA-binding proteins [25, 39, 51].

Next, we determined the total RNA concentrations in the different body fluids. Additional file 1: Fig. S5 illustrates the RNA-content of whole serum (64 ± 16,6 ng / ml serum) and serum-derived EV (9 ± 3,5 ng / ml serum) as well as the RNA content of whole CSF (34,7 ± 11,4 ng / 36 ml CSF) and CSF-derived EV (19,2 ± 4,5 ng / 36 ml CSF) of all samples without obvious blood contamination. Based on these numbers, an average concentration of RNA in whole CSF of approximately 1 ± 0.3 ng per ml CSF can be calculated, which is in accordance with Otake et al. [26], but in contrast to others [7, 30]; the RNA content of EV from 1 ml CSF is 0,5 ± 0,1 ng and thus approximately half as much as seen in whole CSF.

RNA gel analysis of all CSF and serum samples used in this study (depicted in Additional file 1: Fig. S6) and their respective Fragment Analyzer runs (shown in Additional file 1: Fig. S7, S8) revealed obvious differences in the patterns of RNA derived from EV and whole body fluids. The low molecular weight bands in samples from whole CSF and whole serum are more intense and in whole CSF slightly smaller than the RNA from CSF-derived EV. Furthermore, comparisons of the Fragment Analyzer electropherograms, gel analysis and measurements of RNA concentrations of blood-contaminated and normal CSF samples (Additional file 1: Fig. S9) show marked differences in respect to peak size, peak appearance, nucleotide size of the peaks, and RNA yield. Electropherograms from whole CSF samples without blood contamination do not display significant peaks beyond the size of 200 nucleotides.

In the Venn diagrams in Additional file 1: Fig. S10, we compare the NGS patterns of small RNA and long RNA preparations of CSF-, blood-contaminated CSF-, and pure serum-fractions. For the Venn diagrams the average normalized expression of each RNA transcript was calculated, the transcripts were then grouped into low expressed (those with expressions under 33 percentile of average normalised expressions in this group), medium expressed (between 33 and 66 percentile expressions) and highly expressed transcripts (over 66 percentile).The data revealed, that low expression transcripts of blood-contaminated CSF are mainly restricted to blood-contaminated CSF fractions, and only a few of these low expression transcripts are shared with CSF or serum fractions. In contrast, transcripts of blood-contaminated CSF with high expression levels are to a wide extend also found in CSF and/or serum fractions. This distribution implies that contamination of body fluids with blood-derived transcripts can bias NGS patterns of diagnostic samples of CSF and serum fractions in two ways. First, blood-derived transcripts with low expression might mimic the appearance of extraneous transcripts in the body fluids, and second, already small contaminations with blood-derived high expression transcripts could confound NGS-patterns in general. This underlines the urgent need to avoid any blood contamination of samples for meaningful and convincing NGS-based diagnostics.

### Results from small RNA sequence analysis

**Fig. 1:**
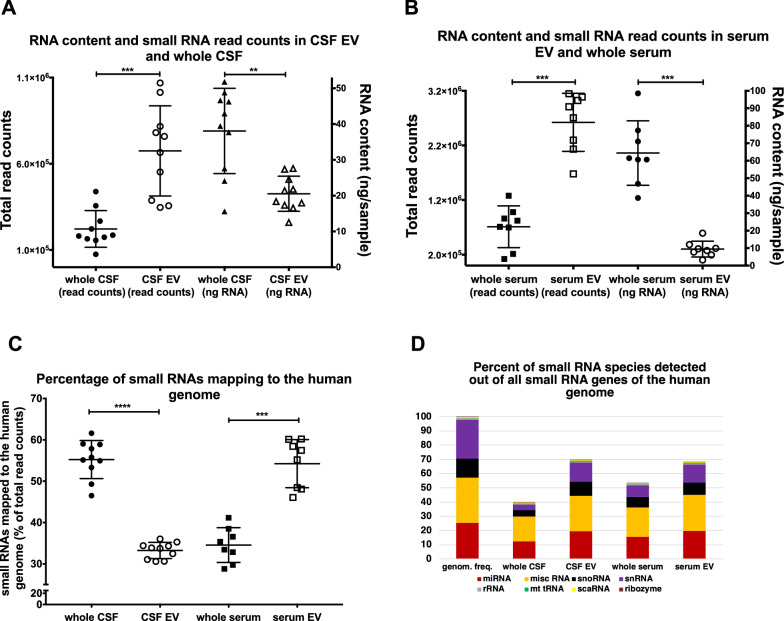
Read counts, RNA content, percentage of small RNAs mapping to the human genome, and percentage of small RNA species detected. Figure **A** depicts the read counts and mean ± S.D. of samples derived from CSF (black full circles, left y-axis) and CSF-derived EV (black empty circles, left y-axis), as well as the RNA content and mean ± S.D. of the same samples (black full triangles represent whole CSF and black empty triangles represent CSF-EV, both right y-axis). Figure **B** shows the read counts with mean ± S.D. of samples derived from RNA of whole serum (black full squares, left y-axis) and serum-derived EV (black empty squares, left y-axis), as well as the RNA content with mean ± S.D. of the same samples (whole serum samples are shown as full black circles and serum EV samples are shown as black empty circles, both right y-axis). Figure **C** displays the percentage of small RNAs mapping to the human genome. Samples from whole CSF (full black circles), from CSF-derived EV (black empty circles), from whole serum (full black squares) and from serum-derived EV (black empty squares) ± their S.D. are shown. The levels of significance are given (****p < 0,0001, ***p < 0,0005, **p < 0,005, two-tailed Mann Whitney test). Figure **D** displays the percentage of small RNA species detected out of all known small RNA genes of the human genome

A comparison of RNA-concentrations and read counts in all four small RNA fractions (Fig. 1A, B) shows an inverse correlation; although the total amount of RNA extracted from EV is significantly less than the amount of RNA directly extracted from the respective whole body fluid, the small RNA total read counts are significantly higher in RNA derived from EV than in the respective whole body fluid. The percentage of small RNAs mapping to the human genome is app. 40% less in CSF-derived EV than in whole CSF, but it is 36% higher in serum-derived EV than in whole serum (Fig. 1C). The partially low alignment rates are similar to reports by others [19, 52], but in contrast to Godoy et al. [52]; the contamination rate according to Kraken [53] is less than 5% in each sample analyzed. The percentage of small RNA species detected out of all small RNA genes of the human genome is higher in EV fractions than in the respective whole body fluids. (Fig. 1D).

**Fig. 2:**
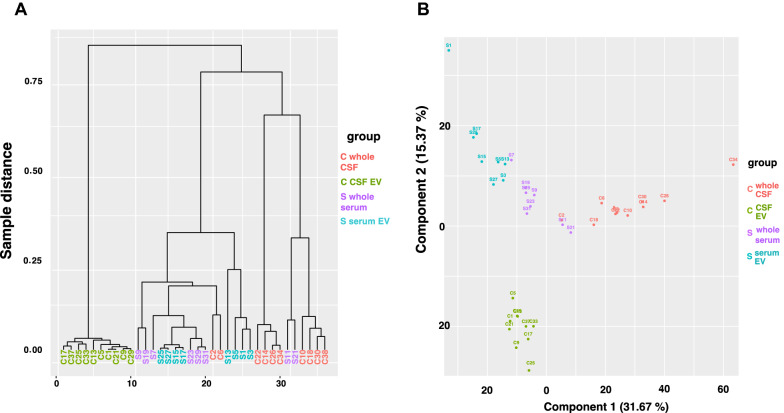
Clustering and PCA plot of small RNA samples. Figure **A** depicts a clustering plot that indicates how well samples cluster together based on the distances between their small RNA NGS profiles. The y-axis shows the sample distance based on the small RNA NGS profiles of the samples; the x-axis indicates the number of samples. Figure **B** displays a two-dimensional PCA plot based on the sequencing profiles of the samples. Each point and number represent a single sample. Light red represents samples from whole CSF, light green colored dots and numbers represents samples from CSF-derived EV, purple represents samples from whole serum, and light blue represents samples from serum-derived EV

**Fig. 3:**
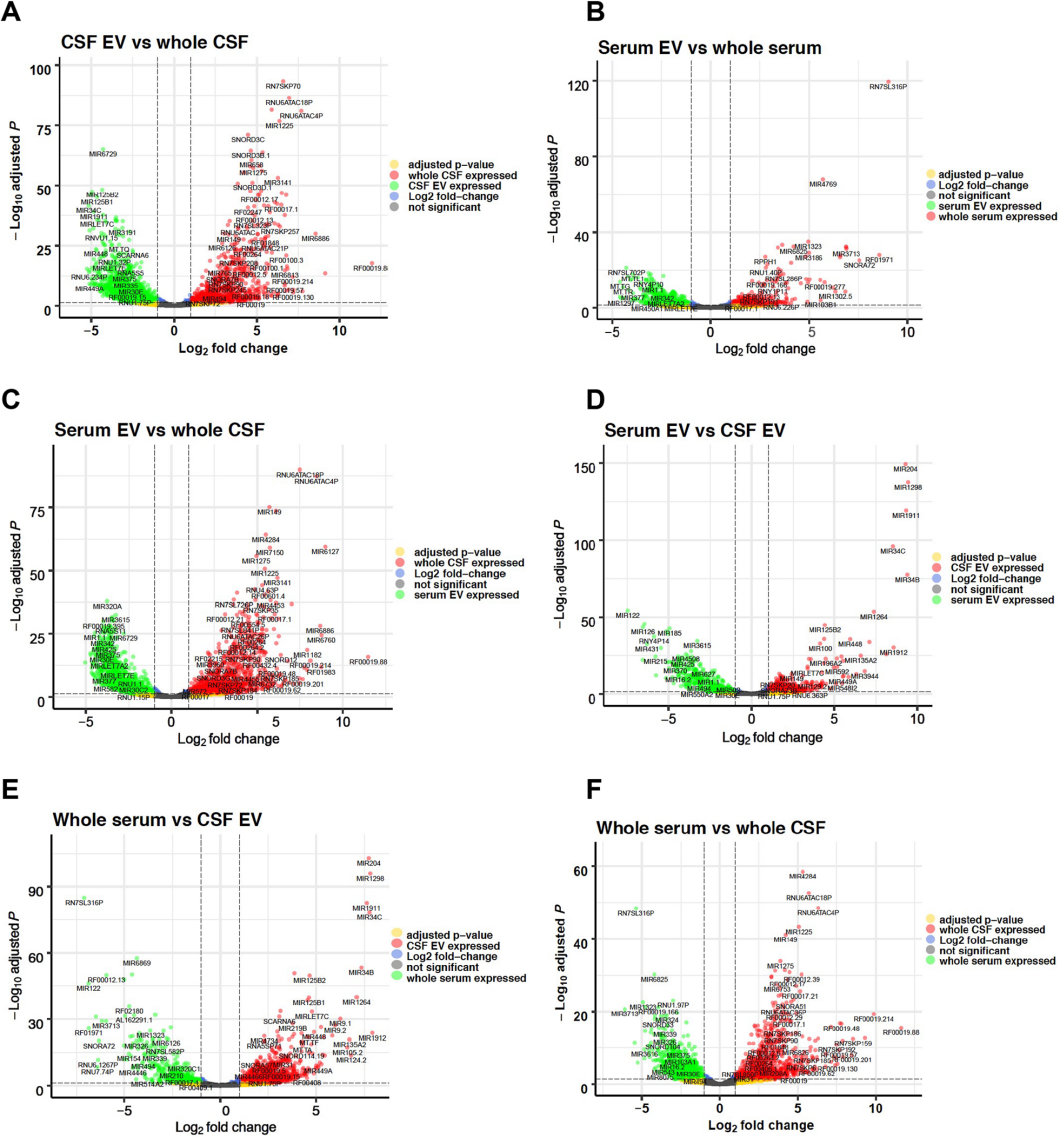
Volcano plots of small RNA samples. The figure shows volcano plots comparing log_2_fold-changes of read counts (x-axis) to -log_10_ of the corresponding adjusted p-values (y-axis) for all small RNA read counts in each comparison. The colors of the dots indicate for the respective small RNA whether the number of read counts exceeds a log_2_fold-change threshold of smaller than − 2 or larger than 2, respectively, represented by the dashed vertical lines, and whether the adjusted p-value of the − log_10_ is larger than 1,30103 (horizontal dashed line, p ≤ 0,05). Grey dots represent read counts of small RNAs with adjusted p-value > 0.05 and a log_2_-change ≤ 2 and ≥ -2 (not significant), green dots represent read counts with log_2_fold-changes of ≤ -2 and an adjusted *p*-value of p ≤ 0.05, blue dots show read counts with log_2_fold-change of ≤ 2 and ≥ -2 but an adjusted p-value of p ≤ 0,05, red dots display read counts with log_2_fold-change ≥ 2 and an adjusted p-value of p ≤ 0,05, yellow dots display read counts with a log_2_fold change ≤ -2 or ≥ 2 but an adjusted *p*-value of p > 0,05 (not significant). Figure **A** shows the comparison of CSF EV versus whole CSF, figure **B** compares serum EV and whole serum, figure **C** serum EV and whole CSF, figure **D** serum EV and CSF EV, figure **E** whole serum and CSF EV, and figure **F** whole serum versus whole CSF. Altogether 4601 variables were analyzed in each plot

**Fig. 4:**
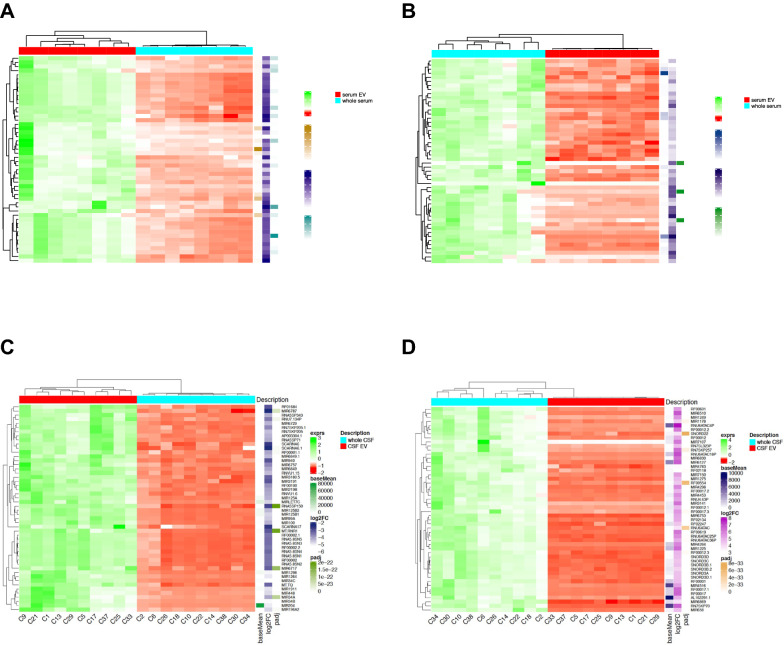
Heat maps of read counts of small RNAs. The figure shows the heat maps comparing the read counts of small RNAs derived from serum EV and whole serum (figure **A** and **B**) and small RNA derived from CSF EV and whole CSF (figure **C** and **D**). The 50 most down-regulated small RNAs (figure **A** and **C**), and the 50 most upregulated (figure **B** and **D**) small RNAs in whole serum and whole CSF are displayed. Dendrograms to the right of the heatmaps cluster the RNA species, dendrograms on top of the heat maps display the clustering of the samples; underneath the heat maps the sample code is depicted. More heat maps comparing further pairs of comparisons are shown in Additional file 1: Fig. S8.

The clustering-plot and the 2-dimensional PCA plot (shown in Fig. 2) indicate how well samples from each group cluster together based on the distances between their small RNA profiles. The well-defined differences between the four analyzed groups are further highlighted by the volcano plots shown in Fig. 3, the heat maps of the top 50 most up- and downregulated genes between two different conditions (Fig. 4 and Additional file 1: Fig. S11), as well as by the number of differentially expressed small RNA transcripts listed in Table 1 (and Additional file 2: Tables S1–S6). Remarkably, among all pairs of groups the largest difference in profiles of 40,4% up- and 18,7% down-regulated small RNA was seen between whole CSF and CSF-derived EV, whereas the smallest difference of all comparisons of 14,7% up- and 9,3% down-regulated transcripts was seen between whole serum and serum-derived EV.

**Table 1:** Significantly up- and down-regulated small RNAs in each pair of conditions

	Serum EV	Whole serum	CSF EV	Whole CSF
Serum EV	–	677/429	441/993	1338/1071
Whole serum	14.71%/9.32%	–	497/1290	968/912
CSF EV	9.58%/21.58%	10.80%/28.04%	–	1858/858
Whole CSF	29.08%/23.28%	21.04%/19.82%	40.38%/18.65%	–

The table summarizes in the upper right part the number of significantly up/down regulated small RNAs in each pair of conditions; the first number shows the transcripts up-regulated in the column group, the second number shows the transcripts up-regulated in the row group. The lower left of the table displays the respective percentages in respect to the total number of 4601 analyzed small RNAs

**Fig. 5:**
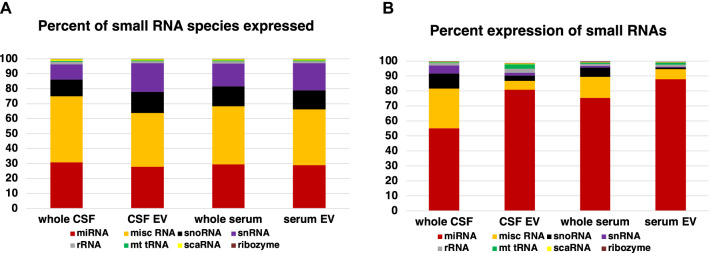
Number of species and average expression level of small RNA. Figure **A** displays the number of transcripts detected in each small RNA family in all four fractions analyzed with NGS. 100% of the y-axis corresponds to all RNA transcripts detected in one fraction. Figure **B** illustrates the average expression levels of small RNA transcripts in each category in the four different fractions tested (based on the number of read counts associated with a small RNA biotype). The y-axis depicts the percentage of total read counts. Altogether 4601 small RNA transcripts were analyzed

More than 96% of all small RNA transcripts expressed in serum and CSF fractions gathered by our small RNA sequencing approach belong to four categories, and in all four collected fractions miscellaneous (misc) RNA and miR comprise approximately two thirds of all detected species (Fig. 5A). Whereas the percentage of small RNA transcripts belonging to a specific small RNA category is rather similar in all four fractions (Fig. 5A); the highest percentage expression in all four fractions is clearly seen with the family of miR, but there are also obvious differences in the relative expression level of misc RNA- and small nucleolar (snoRNA) (Fig. 5B).

**Fig. 6:**
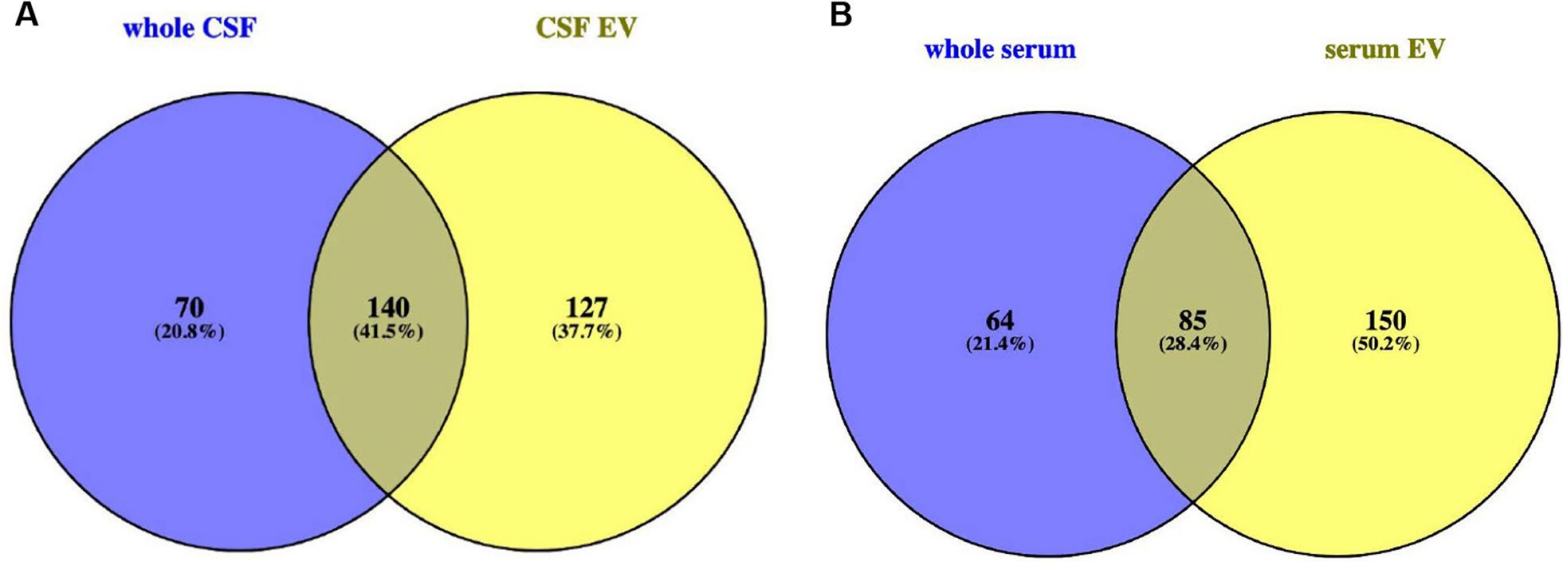
Venn diagrams of WebGestalt analysis with ORA. The Venn diagram in figure **A** shows the difference in sets of transcripts (that govern pathways, cellular and molecular functions and other biological processes) targeted by miR in whole CSF (blue circle) and CSF-derived EV (yellow circle). The Venn diagram in figure B displays the same information for whole serum versus serum-derived EV. The WebGestalt analysis was performed with the ORA enrichment method. Only gene sets significantly different in both, FDR < 0.05 and p < 0.05, are depicted. For further information please also refer to Additional file 3: Tables S7 and S8

MiR act concerted, i.e. a single miR can target different transcripts and a single transcript can be targeted by various miR. As miR are known to be regulators of metabolism and are linked to human diseases [54, 55], we analyzed the potential of the different compositions of miR in EV and body fluids, to influence the expression of target transcripts that govern pathways, cellular and molecular functions and other biological processes with WebGestalt analysis [56]. The Go slim summaries (Additional file 1: Fig. S12) provided with the WebGestalt analysis show the distribution of these target transcripts to subgroups in functional categories that are preselected by the Gene Ontology Consortium [57]. The absolute number of transcripts matching to the subgroups of these functional categories varies between the four analyzed groups of our query due to the different numbers of unambiguously mapped entrezgene ID in the query list (interesting list), but relative numbers, i.e. the number of matches in one subgroup in respect to all unique matches found in one category are very similar. The latter can be explained by the concerted action of miR that causes a significant overlap of targets for different miR and the fact that differentially targeted transcripts are part of the same subgroup in a functional category. Nevertheless, the over-representation analysis (ORA) of WebGestalt reveals that different compositions of miR in EV and respective whole body fluid have the capability to affect different sets of target transcripts. The Venn diagrams in Fig. 6 (and Additional file 3: Tables S7, S8) show obvious differences in those transcript sets that are potentially targeted by miR enriched in whole CSF and those enriched in CSF-derived EV. From altogether 337 characterized categories of transcript sets, 37,7% are likely to be targeted only by CSF-derived EV and 20,8% only by miR found in whole CSF, whereas 41,5% are targeted from miR in both fractions. Similarly, both serum fractions share less than 29% from 299 identified targeted transcript sets, whereas more than 50% are potentially targeted only by miR derived from serum EV and more than 21% are potentially affected by miR found in whole serum.

### Results from long RNA sequence analysis

**Fig. 7:**
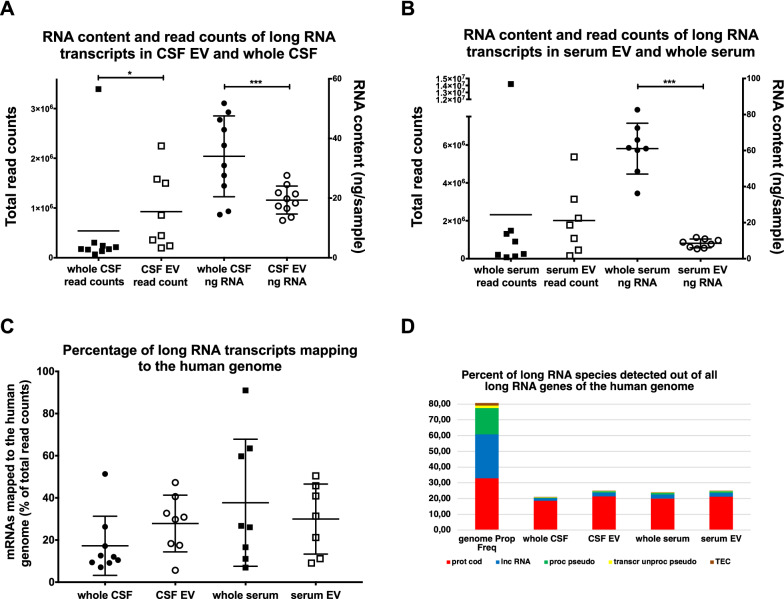
Read counts, RNA content, long RNA transcripts mapping to the human genome, and percentage of long RNA species detected. Figure **A** depicts the read counts of long RNA transcripts and mean ± S.D. of samples derived from of whole CSF (black full squares, left y-axis) and CSF-derived EV (black empty squares, left y-axis), as well as the RNA content and mean ± S.D. of the same samples (black full circles represent whole CSF and black empty circles represent CSF-EV, both right y-axis). Figure **B** shows the read counts of long RNA transcripts with mean ± S.D. of samples derived from whole serum (black full squares, left y-axis) and serum-derived EV (black empty squares, left y-axis), as well as the RNA content with mean ± S.D. of the same samples (whole serum samples are shown as full black circles and serum EV samples are shown as black empty circles, both right y-axis). Figure **C** displays the percentage of long RNA transcripts mapping to the human genome. Samples from whole CSF (full black circles), from CSF-derived EV (black empty circles), from whole serum (full black squares) and from serum-derived EV (black empty circles) ± their S.D. are shown. The levels of significance are given (***p < 0,0005, *p < 0,05, two-tailed Mann Whitney test). Figure **D** displays the percentage of long RNA transcripts belonging to a long RNA family in respect to all known long RNA genes of the human genome (first column with more than 80%); please mind that only the five most abundant long RNA families are displayed (therefore, the y-axis is scaled to 80%). Abbreviations: prot cod, protein coding; lnc RNA, long non-coding RNA; proc pseudo, processed pseudogene; trans unproc pseud, translated unprocessed pseudogene; TEC, Tyrosine-protein kinase Tec

Similar to the small RNA fractions, Fig. 7A, B depict that the total amounts of RNA extracted from CSF- and serum-EV fractions for long RNA analysis are significantly less than the amounts of RNA directly extracted from the respective body fluids, but the average total read counts are quite similar. In contrast to small RNA, the percentage of long RNA transcripts mapping to the human genome has a high variation in each of the four fractions. The number of RNAs mapping to the human genome can vary up to one order of magnitude in one fraction, and there is no significant difference in the percentage of mapping between the four groups (Fig. 7C). Similar to small RNAs, the relatively low and variable alignment rates remain unexplained. The percentage of long RNA species detected out of all long RNA genes of the human genome is similar in both serum fractions, but differs in CSF fractions, where whole CSF has approximately 3% less protein coding RNAs (p < 0,005) as compared to CSF-derived EV (Fig. 7D).

**Fig. 8:**
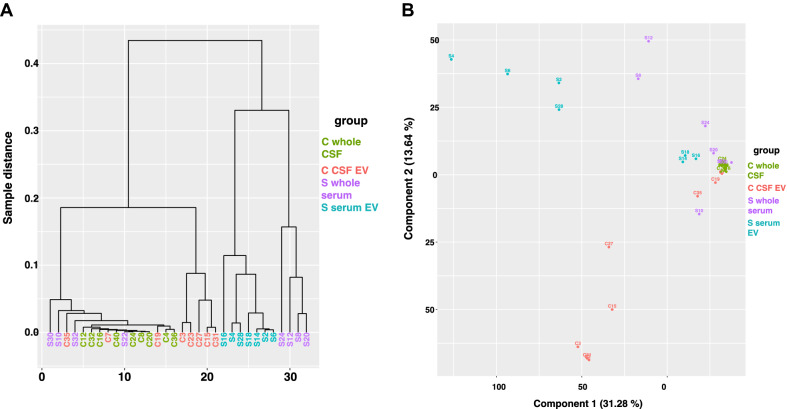
Clustering and PCA plot of long RNA transcripts in all samples. Figure **A** depicts a clustering plot that indicates how well samples cluster together based on the distances between their long RNA NGS profiles. The y-axis shows the sample distance based on the long RNA NGS profiles of the samples; the x-axis indicates the number of samples. Figure **B** displays a two-dimensional PCA plot based on the sequencing profiles of the samples. Each point and number represent a single RNA sample. Green colored dots and numbers represents samples from whole CSF, light red represents samples from CSF-derived EV, purple represents samples from whole serum, and light blue represents samples from serum-derived EV

**Fig. 9:**
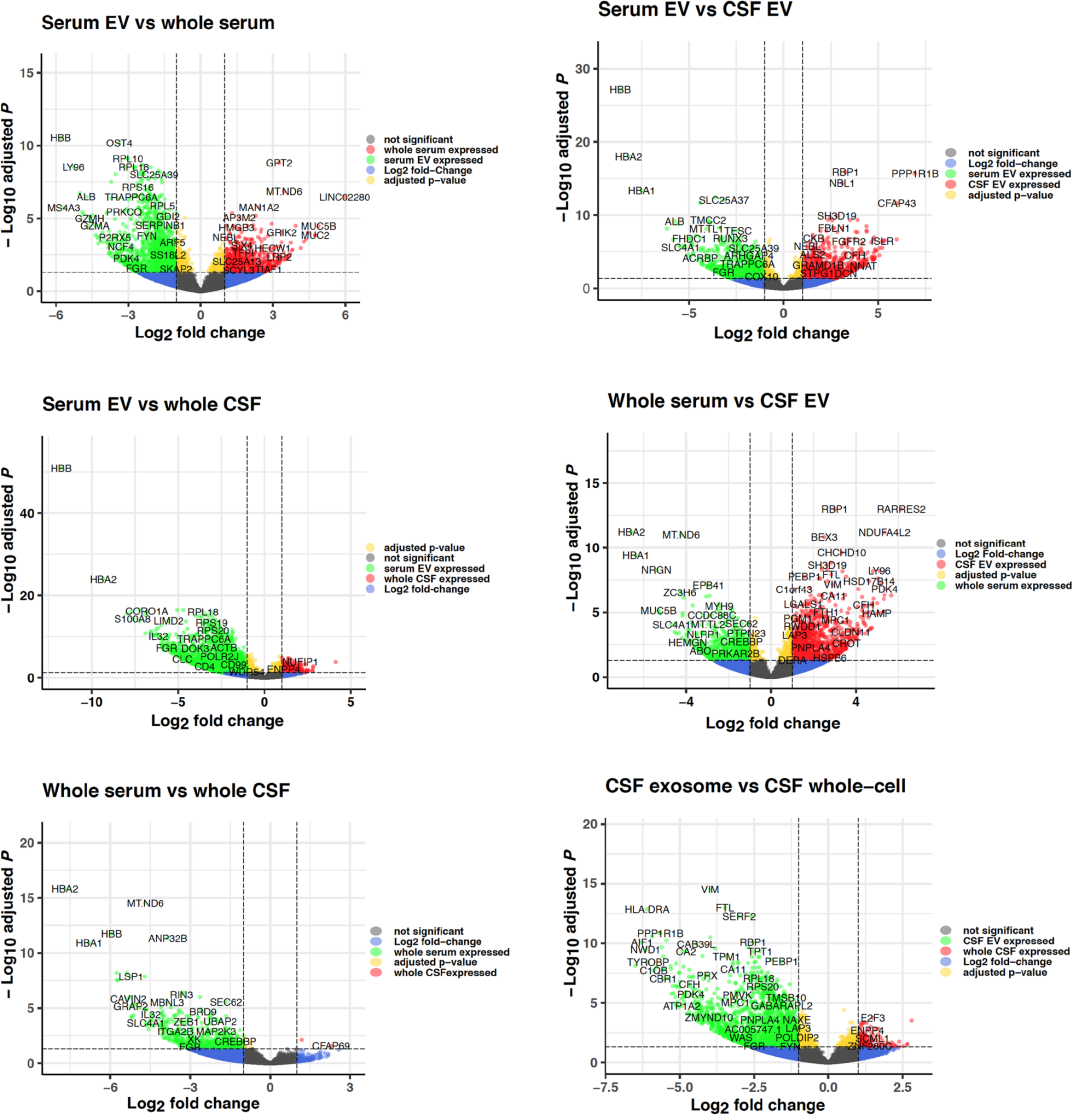
Volcano plots of long RNA transcripts. The figure shows volcano plots comparing log_2_fold-changes of read counts (x-axis) to -log_10_ of the corresponding adjusted p-values (y-axis) for all long RNA read counts in each comparison. The colors of the dots indicate for the respective long RNA whether the number of read counts exceeds a log_2_fold-change threshold of smaller than − 2 or larger than 2, respectively, represented by the dashed vertical lines, and whether the adjusted p-value of the − log_10_ is larger than 1,30,103 (horizontal dashed line, p ≤ 0,05). Grey dots represent read counts of long RNAs with adjusted p-value > 0.05 and a log_2_-change ≤ 2 and ≥ -2 (not significant), green dots represent read counts with log_2_fold-changes of ≤ − 2 and an adjusted *p*-value of p ≤ 0.05, blue dots show read counts with log_2_fold-change of ≤ 2 and ≥ -2 but an adjusted p-value of p ≤ 0.05, red dots display read counts with log_2_fold-change ≥ 2 and an adjusted p-value of p ≤ 0.05, yellow dots display read counts with a log_2_fold change ≤ -2 or ≥ 2 but and an adjusted p-value of p > 0.05 (not significant). Figure **A** compares serum EV and whole serum, figure **B** serum EV and CSF EV, figure **C** serum EV and whole CSF, figure **D** whole serum and CSF EV, figure **E** whole serum and whole CSF, and figure F CSF EV and whole CSF. Altogether 20,686 variables were analyzed in each plot

The clustering- and the two-dimensional PCA plot (shown in Fig. 8) of analyzed long RNA transcripts in the all samples indicate that long RNA profiles cluster less well together than the corresponding small RNA profiles. Differences between the four analyzed groups are still obvious as seen by the volcano plots shown in Fig. 9, the heat maps of the top 50 most up- and downregulated genes (Additional file 1: Fig. S13), and the number of differentially expressed long RNA transcripts listed in Table 2 (and Additional file 4: Tables S9–S14). Albeit the percent differences of long RNA transcripts among all pairs of groups are relatively small as compared to the small RNA NGS profiling, the absolute numbers of differentially expressed transcripts depend on the pairing of the comparison and range from 2 to 2535 significantly differentially expressed long RNA. The largest difference in NGS profiles of 12,25% up- and 2,53% down-regulated long RNA transcripts is seen between serum EV and whole CSF, the second largest between CSF EV and whole CSF (9,48% up- 0,78 down-regulated), whereas almost all transcripts found in whole CSF are also detected in whole serum. Table 2, heat maps und volcano plots reveal that most long RNA transcripts detected in whole CSF are also found in all three other fractions.

**Table 2:** Significantly up- and down-regulated long RNAs in each pair of conditions

	Serum EV	Whole serum	CSF EV	Whole CSF
Serum EV	–	1290/763	936/887	2535/524
Whole serum	6.24%/3.69%	–	629/1188	571/2
CSF EV	4.52%/4.29%	3.04%/5.74%	–	1960/161
Whole CSF	12.25%/2.53%	2.76%/0.01%	9.48%/0.78%	–

The table summarizes in the upper right part the number of significantly up/down regulated long RNAs in each pair of conditions; the first number shows the transcripts up-regulated in the column group, the second number shows the transcripts up-regulated in the row group. The lower left of the table displays the respective percentages in respect to the total number of 20,686 analyzed long RNAs

**Fig. 10:**
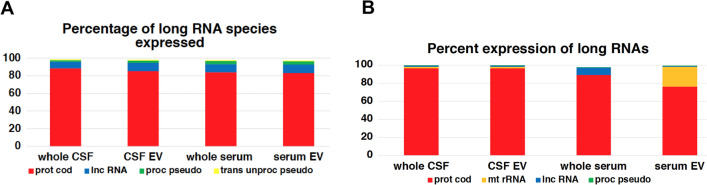
Number of species and average expression levels of long RNA species. Figure **A** displays the number of species detected in each long RNA family in all four fractions analyzed with NGS. 100% of the y-axis corresponds to all RNA species detected in one fraction (only the four most abundant long RNA species are shown). Figure **B** illustrates the average expression levels of transcripts of long RNA species in the four different fractions tested (based on the number of read counts associated with a small RNA biotype). The y-axis depicts the percentage of total read counts. Altogether 20,686 long RNA transcripts were analyzed (only transcripts of the four most abundant species are shown). Prot cod, protein coding; mt rRNA, mitochondrial ribosomal; mt tRNA, mitochondrial transfer RNA; lnc, long non-coding; proc pseudo, processed pseudogene; trans unproc pseudo, translated unprocessed pseudogene

More than 98% of all long RNA species expressed in blood and CSF fractions gathered by our small RNA sequencing approach belong to four categories, and in all four fractions the protein coding RNA comprise more than 80% of all detected species. Albeit the percentage of each specific long RNA family is rather similar in all four fractions, the percent expression of long RNA, i.e. the number of read counts associated with a specific long RNA family, differs and is most obvious between CSF and serum fractions, but also apparent between whole serum and serum EV (Fig. 10A and B).

**Fig. 11:**
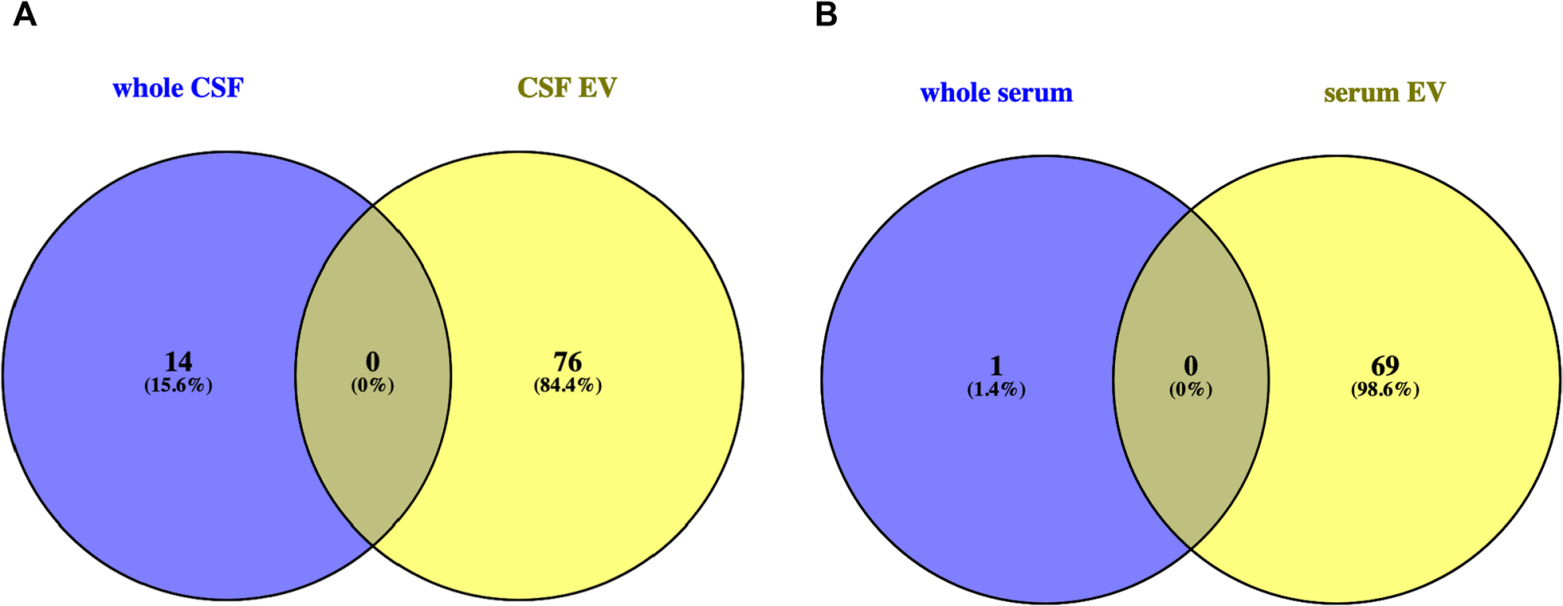
Venn diagrams of WebGestalt analysis from mRNA with Over-Representation Analysis. The Venn diagram in figure **A** shows the difference in sets of mRNA transcripts (that govern pathways, cellular and molecular functions and other biological processes) expressed in whole CSF (blue circle) and CSF-derived EV (yellow circle). The Venn diagram in figure B displays the same information for whole serum versus serum-derived EV. The WebGestalt analysis was performed with the ORA enrichment method. Only gene sets significantly different in both, FDR < 0.05 and p < 0.05 are depicted. For further information please also refer to Additional file 5: Tables S15 and S16

Grouping of the long RNA transcripts by WebGestalt in transcript sets directly regulating categories of biological processes revealed less differentially expressed sets of transcripts in all four fractions as compared to the number of possible gene sets indirectly affected by miR. Most categorized transcripts in serum and CSF fractions are found in the respective EV fractions; in serum almost 99% and in CSF more than 84% of significantly differentially expressed transcripts are found in EV (Fig. 11 and Additional file 5: Table S15 and S16). The strong bias for accumulation of long RNA transcripts in EV might be explained by the fact that long transcripts in body fluids not protected by membranes or protein binding are prone to degradation by RNA degrading enzymes.

## Discussion

A major obstacle for reliable analysis of RNA profiles from human CSF, unlike serum, is the limited sample volumes of CSF usually provided by clinical diagnostics, as well as the very low RNA content of CSF [19, 22, 26, 27]. Furthermore, quality checks of CSF-derived RNA are difficult and often RNA peaks are hardly seen in electropherograms of analyzers [26, 58]. Additionally, minor amounts of RNA can significantly decrease the number of robustly detected RNA species in analysis [26], and natural variations of donors can confound statistical analysis [14, 30]. These circumstances make definite and consistent measurements of RNA concentrations difficult. More reliable RNA-profiling should be possible from larger volumes of pooled CSF samples [58]. In order to provide a comprehensive and unequivocal analysis of small and long RNA profiles of whole CSF and CSF-derived EV, we used outsized volumes of CSF prepared from pooled CSF of healthy male and female donors aged between 0,4 and 93,4 years, that should level out natural variations, and thus to determine the fundamental characteristics of RNA distribution in human CSF to set a solid scientific basis for future studies employing also smaller CSF volumes.

As access to human CSF depends on the clinical supply and is usually only provided after the end of routine diagnostics, we first examined whether handling during routine diagnostics might affect the RNA content of CSF. Similar to blood, serum, and plasma [15, 24, 25, 32, 38] we show, that the extracellular RNA content of CSF is neither affected by long time storage at 4 °C nor by RNAse treatment. On the other hand, we show that the exclusion of contamination by foreign RNA as for example from blood-derived cells, is an important premise for meaningful and convincing RNA profiling of human CSF samples, as even small contaminations can cause significant bias of the profiles. This is obvious from our electropherograms, RNA measurements, gel analysis and Venn diagrams comparing transcripts of blood-contaminated CSF samples with respective CSF and serum samples, as well as from observations by others [15, 18, 20, 28, 37, 51, 58, 59].

All analysis of the small RNA-profiling, show the disparity of the RNA content of each of the four different fractions analyzed, and surprisingly the largest difference (altogether 59%; Table 1) between two groups is not seen between a serum and a CSF fraction, but between whole CSF and CSF-derived EV, whereas the smallest difference is seen between whole serum and serum EV (24%) and the second smallest between serum EV and CSF EV (31,2%). The volcano plot of serum EV versus CSF EV in Fig. 3 has few points with very small p-values representing strongly differentially regulated transcripts that contribute to the distinct patterning in the clustering and PCA plot, but the general profile of the volcano plot from both EV fractions is similar flat as the plot of serum EV versus whole serum and thus obviously different to the volcano plots of the remaining four comparisons. Furthermore, Fig. 1D points to an obvious similarity of both EV fractions in respect of the percentage of small RNA species detected out of all known small RNA genes of the human genome. In addition, Venn diagrams of all significantly expressed small RNA and of all significantly expressed miR in each of the four fractions shows the most common transcripts between both EV fractions. Moreover, the WebGestalt analysis also reveals the largest number and percentage of commonly affected sets of target-transcripts by miR expressed in both EV fractions; i.e. in these respects the two EV fractions are even more similar than the two serum fractions (Additional file 1: Fig. S14). These data point to an exchange of small RNA between serum and CSF via EV, an assumption supported by recently accumulated evidence suggesting that EV can cross the blood–brain barrier [60, 61]. As the difference in small RNA content between serum fractions is the lowest whereas it is the highest between CSF fractions, it is likely that the traffic of EV is mainly from serum to CSF and not vice versa. If RNA is exchanged between serum and CSF, the measurement of transcripts in only one EV fraction could be misleading for diagnostics, and a ratio of respective serum and CSF fractions, as used for proteins in CSF diagnostics [62], would be more appropriate and possibly informative in respect to the integrity of the blood–brain barrier.

A graphical evaluation of 664 small RNAs significantly up- or down-regulated in all four fractions (Additional file 1: Fig. S15) shows that most of the small RNAs have equivalent concentrations, in both, body fluid and corresponding EV, but some show a reciprocal pattern, i.e., have higher read counts in CSF than in serum, and are less expressed in CSF-derived EV than in serum-derived EV. This pattern can neither be explained by passive diffusion across the blood–brain barrier nor by a cell homeostasis-driven, constitutive and proportionate release of small RNAs from cells by EV, nor by constitutive non-vesicular pathways into the corresponding body fluids. These inverse expression levels of some small RNAs in body fluid and corresponding EV are more likely due to a general or cell-specific sorting mechanism of small RNAs, or possibly facilitated by a selective transport of certain EV across the blood–brain barrier.

The WebGestalt analysis in our study shows, that miR, significantly differentially expressed in EV and respective body fluid, have the potential to affect different sets of transcripts and thus different pathways and distinctive cellular, molecular and biological functions. Therefore, miR and possibly other small RNAs in EV and the respective body fluid, might also have the potential to differentially interfere with the development and prevention of human diseases. A direct comparison of WebGestalt-miR target sets between whole CSF and CSF derived EV shows that miR-targed gene sets involved in neurological development and diseases are differentially represented in each fraction. E.g., whereas in CSF derived EV the analysis revealed four differentially expressed sets of miR targets (involved in central nervous system neuron differentiation, neuron projection guidance, postsynaptic specialization, and regulation of commissural axon pathfinding by SLIT and ROBO), there are eight different sets of differentially expressed miR targets in whole CSF (involved in amyloid-beta metabolic process, loss of function of MECP2 in Rett syndrome, neural precursor cell proliferation, neurodegenerative diseases, neuron to neuron synapse, regulation of synapse structure or activity, Sema4D induced cell migration and growth-cone collapse, and synaptic vesicle cycle) (Additional file 3: Table S7).This underlines that searches for diagnostic small RNA markers might easily fail, if transcripts expressed in a disregarded fraction are not taken into account. It is conceivable that the expression level of a given small RNA differs between diseased and healthy people in only one compartment (body fluid or EV) but not in the other, and therefore, comprehensive searches for small RNA-based disease markers ought not be restricted to either the body fluid or the body fluid-derived EV, but should rather encompass both fractions. This holds true for serum as well, although the difference in small RNA content between serum and serum-derived EV is less than half of the difference between whole CSF and CSF-derived EV (Table 1).

The differences in the long RNA-profiling seem less profound than the small RNA-profiling, and most long RNA transcripts found in whole CSF are also found in the three other fractions. Nevertheless, the generally higher number of significantly differentially expressed long RNAs leads to comparable total numbers of up- and down-regulated transcripts in both preparations (Table 2), and thus, the value of long transcripts for searches of molecular disease markers should not be underestimated. E.g., a direct comparison of WebGestalt mRNA sets between whole CSF and CSF derived EV shows that coding transcripts involved in neurological development and diseases are also differentially represented in whole CSF and CSF derived EV. In CSF derived EV the WebGestalt analysis revealed three differentially expressed long RNA transcript sets (involved in Alzheimer disease, Huntington disease, neural nucleus development) and six long RNA transcript sets in mitochondrial metabolism (mitochondrial inner membrane, mitochondrial membrane part, mitochondrial protein complex, mitochondrial protein import, mitochondrial translation, mitochondrial transport), whereas no such transcript sets are found in the whole CSF fraction (Additional file 5: Table S15). Again, the WebGestalt analysis of differentially expressed long RNA transcripts in EV and respective body fluid, have the potential to unequally affect cellular and biological processes and hence also might differentially interfere with the development and prevention of human diseases.

## Conclusions

This study shows that the composition of small and long RNA differs significantly between whole body fluid and its respective EV fraction. Differentially expressed long RNAs belong to different transcript sets involved in distinctive biological functions, and differentially expressed miR can target specific transcripts specific to different cellular and molecular functions. We show that trace amounts of blood-derived contaminations of CSF can bias RNA-based CSF diagnostics and the presented data imply a transport of EV from serum to CSF across the blood–brain barrier. These aspects are important for the search of RNA-based diagnostic markers from CSF and serum. A future collaboration of hospitals with access to CSF analysis could allow the establishment of age- and sex-dependent standard RNA patterns for CSF diagnostics, similar and in addition to the already established protein patterns.

## Supplementary Information

**Additional file 1:** Age distribution of patients, volume size, and erythrocyte and leucocyte numbers of CSF and serum samples. Fig. S2 RNA content of 100 KDa column concentrates and filtrates. Fig. S3 Age- and sex-distribution of serum samples. Fig. S4 RNA content of CSF samples in dependence of the storage temperature and RNAse treatment. Fig. S5 RNA content of CSF and serum samples. Fig. S6 Gel analysis of all RNA samples. Fig. S7 Electropherogramms of the RNA samples from serum. Fig. S8 Electropherogramms of the RNA samples from CSF. Fig. S9 Comparison of RNA content and electropherogramms of the RNA samples from CSF and blood-contaminated CSF. Fig. S10 Venn diagrams and UpSet plots form small and long RNA. Fig. S11 Heat maps of small RNA read counts not shown in figure 4. Fig. S12 GO Slim summaries of miR targets from whole CSF, CSF-EV, whole serum, and serum EV. Fig. S13 Heat maps of read counts encompassing all possible comparisons of down- and up-regulated long RNA transcripts. Fig. S14 Venn diagrams of significantly expressed small RNA transcripts, expressed miR, and target transcripts of expressed miR. Fig. S15 Distribution of small RNAs in body fluids and their respective EV.

**Additional file 2:** Read counts of differentially expressed small RNA transcripts listed in table1.

**Additional file 3:** Target sets of miR expressed in serum and CSF fractions

**Additional file 4:** Read counts of differentially expressed long RNA transcripts listed in table 2 .

**Additional file 5:** Transcript sets of mRNA expressed in serum and CSF fractions.

## Data Availability

Datasets analysed during the current study not included in this published article are available on reasonable request.

## Abbreviations

CNS: Central nervous system
CSF: Cerebrospinal fluid
EV: Extracellular vesicles
Mir: MicroRNA
NGS: Next generation sequencing
PCA plot: Principal component analysis plot
