# Supplementary material for: Small and long RNA transcriptome of whole human cerebrospinal fluid and serum as compared to their extracellular vesicle fractions reveal profound differences in expression patterns and impacts on biological processes
Source: J Transl Med. 2022 Sep 8;20:413. doi: 10.1186/s12967-022-03612-3 (PMC9461220; doi:10.1186/s12967-022-03612-3)
Supplement: Supplementary file 1 — Additional file 1: Age distribution of patients, volume size, and erythrocyte and leucocyte numbers of CSF and serum samples. Fig. S2 RNA content of 100 KDa column concentrates and filtrates. Fig. S3 Age- and sex-distribution of serum samples. Fig. S4 RNA content of CSF samples in dependence of the storage temperature and RNAse treatment. Fig. S5 RNA content of CSF and serum samples. Fig. S6 Gel analysis of all RNA samples. Fig. S7 Electropherogramms of the RNA samples from serum. Fig. S8 Electropherogramms of the RNA samples from CSF. Fig. S9 Comparison of RNA content and electropherogramms of the RNA samples from CSF and blood-contaminated CSF. Fig. S10 Venn diagrams and UpSet plots form small and long RNA. Fig. S11 Heat maps of small RNA read counts not shown in figure 4. Fig. S12 GO Slim summaries of miR targets from whole CSF, CSF-EV, whole serum, and serum EV. Fig. S13 Heat maps of read counts encompassing all possible comparisons of down- and up-regulated long RNA transcripts. Fig. S14 Venn diagrams of significantly expressed small RNA transcripts, expressed miR, and target transcripts of expressed miR. Fig. S15 Distribution of small RNAs in body fluids and their respective EV. [file 12967_2022_3612_MOESM1_ESM.docx]

**Supplementary figures**

Supplementary figure 1:

Age distribution of patients, volume size, and erythrocyte and leucocyte numbers of CSF and serum samples.

**A**	**B**

**C**	**D**

**E	F**

Figure A shows a scattered dot plot with mean and standard deviation of the age distribution of the patients whose CSF was used for next-generation sequencing. Figure B depicts a box-plot with whiskers of the sample size from the same samples as in A; the middle line in the box represents the median volume, the lower and upper line of the box the 25^th^ and 75^th^ percentile, and the lines below and above the box represent the 10^th^ and 90^th^ percentile. Figure C illustrates the erythrocyte (left y-axis) and leucocyte counts (right y-axis) before centrifugation of the same CSF samples as in A; again box plots with median counts, the 10^th^, 25^th^, 75^th^ and 90^th^ percentile are shown, please mind that some percentiles are not apparent, and both y-axes have two segments. In figure D the scattered dot plot with mean and standard deviation displays the age distribution of the patients whose CSF was contaminated with blood or had high erythrocyte counts before the 105 x g centrifugation step; besides the single values the mean and standard deviation are shown. In figure E the box plot with 10^th^, 25^th^ percentile, median, and 50^th^, 75^th^ and 90^th^ percentile represents the volume distribution of the same blood-contaminated CSF samples as in figure D. Figure F displays the erythrocyte (left y-axis) and leucocyte counts (right y-axis) of the same samples as in figure D before the centrifugation step; please mind that both y-axes have two segments.

Supplementary figure 2:

RNA content of 100 KDa column concentrates and filtrates.

**A**	**B**

Figure A shows a dot plot with means of the RNA content of concentrates (circles) and filtrates (triangles) of human blood-contaminated CSF, Figure B depicts a dot plot with means of the RNA content of concentrates (circles) and filtrates (triangles) of human serum. Concentrates were generated with 100 KDa ultraspin columns; the RNA of the filtrates was extracted by concentrating the 100 KDa filtrates in 3 KDa ultraspin columns, and RNA was then extracted from the 3 DKa concentrates exactly as described for 100 KDa ultraspin columns. The level of significance (**p < 0,005, two-tailed Mann Whitney test) is given.

Supplementary Figure 3:

Age- and sex-distribution of serum samples.

The scattered dot-plot depicts the age- and sex-distribution of the persons who donated serum for next-generation sequencing. The line in the middle shows the mean age of the donors, the upright triangles represent male, the circles female donors.

Supplementary figure 4:

RNA content of CSF samples in dependence of the storage temperature and RNAse treatment.

**A**	**B**

Figure A depicts dot plots with means of the RNA content of CSF samples extracted from concentrates of ultraspin columns; samples were either stored at -80 °C after clinical analysis (circles) or left at 4 °C for 14 days (triangles) until processing. Figure B displays dot plots with means of the RNA content of blood-contaminated CSF samples extracted from concentrates of ultraspin columns; concentrates were either left at 37 °C for 1 h (circles) or treated with RNAse at 37 °C for 1 h before RNA extraction.

Supplementary figure 5:

RNA content of CSF and serum samples.

The figure represents the RNA content in 36 ml CSF samples (full black circles), in CSF-derived EV from 36 ml CSF (empty circles), in 1 ml whole serum samples (full black squares) and in serum-derived EV from 1 ml serum (empty squares). The level of significance (****p < 0,0001, two-tailed Mann Whitney test) is given. Please mind that for the sake of comparable scaling the RNA-content of 36 ml CSF and 1 ml serum are shown.

Supplementary figure 6:

Gel analysis of all RNA samples.

The figure illustrates the gel analysis of the RNA-samples generated from EV of serum (SE_x_ in black writing with small numbers indicating the sample number), from whole serum (SC_x_ in black/green writing), from RNA samples derived from EV of human CSF (CE_x_ in red/black writing), and RNA-samples from column-extracted whole CSF (CC_x_ in red/green writing). Small numbers in blue writing indicate the blood-contaminated CSF-samples (CC_x_ column-extracted blood-contaminated whole CSF, CE_x_ EV from blood-contaminated CSF).

Supplementary figure 7:

Electropherogramms of the RNA samples from serum.

The figure depicts the electropherogramms of the RNA samples derived from EV of human serum (SE_x_ in black writing with small numbers indicating the sample number), and RNA-samples from whole serum (SC_x_ in black/green writing).

Supplementary figure 8:

Electropherogramms of the RNA samples from CSF.

The figure depicts the electropherogramms of the fragment analysis of RNA extracted from EV of human CSF (CE_x_ in red/black writing with small numbers indicating the sample number), and RNA-samples from column-extracted whole CSF (CC_x_ in red/green writing).

Supplementary figure 9:

Comparison of RNA content and electropherogramms of the RNA samples from CSF and blood-contaminated CSF.

**A	B**

Figure A displays the electropherogramms and gel runs of RNA extracted from EV of human blood-contaminated CSF (CE**_b_**_x_ in red/black writing with a “b” in front of the small numbers indicating the sample number), RNA-samples from blood-contaminated, whole CSF (CC**_b_**_x_ in red/green writing), RNA-samples from EV of human CSF without any obvious signs of blood contamination (CE_x_ in red/black writing with small numbers indicating the sample number), and RNA-samples from whole CSF (CC_x_ in red/green writing) without any obvious signs of blood-contamination. Figure B compares the RNA content of all samples of the four CSF groups. The level of significance (** p < 0,01, two-tailed Mann Whitney test) is given.

Supplementary figure 10:

Venn diagrams and UpSet plots form small and long RNA.

**A**

**B**

**C**

**D**

The figure depicts Venn diagrams sorted according to the expression levels of the transcripts of whole (A) and EV fractions (B) of small RNA preparations, and of whole (C) and EV fractions (D) of long RNA preparations, as well as the respective UpSet plots [63] of the comparisons. These diagrams were generated by calculating for each group the average normalized expressions of each transcript. Then in each group the transcripts were divided into low expressed (those with expressions under the 33 percentile of average normalized expression in this group), medium expressed (between the 33 and 66 percentile) and highly expressed (over 66 percentile). Please mind that UpSet plots in figure A and B refer to the same transcript pool and are thus identical; the same applies for C and D.

Supplementary figure 11:

Heat maps of small RNA read counts not shown in figure 4.

**A	B**

**C	D**

**E	F**

**G	H**

The figure shows heat maps encompassing all possible comparisons of small RNA read counts of the four different groups that are not shown in figure 4.

Supplementary figure 12:

GO Slim summaries of miR targets from whole CSF, CSF-EV, whole serum, and serum EV.

**A**

**B**

**C**

**D**

GO Slim summaries of miR targets from whole CSF (A), CSF-EV (B), whole serum (C), and serum EV (D). The GO Slim summaries show the distribution of target transcripts to standard subgroups of functional categories that are annotated by the Gene Ontology Consortium. The y-axis represents the number of transcripts associated with the predetermined standard GO Slim subgroups. The bar graphs contain no statistical information but show a numerical overview of the matching transcripts of our interest list (query list) to the preselected subgroups of the GO consortium.

Supplementary figure 13:

Heat maps of read counts encompassing all possible comparisons of down- and up-regulated long RNA transcripts.

**A	B**

**C	D**

**E	F**

**G	H**

**I	J**

**K	L**

The figure shows heat maps of read counts encompassing all possible comparisons of down- (figure A-F) and up- (figure G-L) regulated long RNA of all four analyzed fractions. Please mind that the comparison of up-regulated long RNAs between whole CSF and whole serum (figure K) revealed only two up-regulated transcripts in whole CSF.

Supplementary figure 14:

Venn diagrams of significantly expressed small RNA transcripts, expressed miR, and target transcripts of expressed miR.

**A	B**

 **C**

The Venn diagram in figure A shows the overlap of significantly expressed small RNA transcripts (based on gene ID) in each of the four fractions analyzed (more than 10 Read Counts in at least one sample of the fraction for both comparisons). The comparison depicted in figure B shows the overlap of significantly expressed miR in all four fractions. The Venn diagram in figure C represents the overlap of target transcripts affected by miR found in each of the four fractions analyzed (wh, whole); only miR were included with adjusted p-values < 0,05 and a log2 fold-change of $\leq-1$and $\geq1$. Target transcripts were determined by WebGestalt analysis and only targets with p < 0,02 and FDR < 0,05 were included in this analysis.

Supplementary figure 15:

Distribution of small RNAs in body fluids and their respective EV.

The figure depicts the relation of small RNAs in whole serum and whole CSF and in serum- and CSF-derived EV. X- and y-axis are scaled to log2-fold; each dot represents a single transcript; for the sake of clarity only one data point in each quadrant is annotated.
